# Precision Medicine in Diabetes, Current Research and Future Perspectives

**DOI:** 10.3390/jpm12081233

**Published:** 2022-07-28

**Authors:** Roberto Franceschi

**Affiliations:** Pediatric Diabetology Unit, S.Chiara Hospital of Trento, Largo Medaglie d’Oro, 9, 38122 Trento, Italy; roberto.franceschi@apss.tn.it; Tel.: +39-0461-903538

The prevalence of diabetes has tripled over the past 2 decades, and by 2050, it is estimated to affect 700 million adults [1]. New drugs and technologies are available for diabetes treatment; however, it is challenging for patients to achieve glycemic targets; a potential solution, to improve outcomes in diabetes care and reduce costs, is to shift towards precision medicine [2].

Recently the American Diabetes Association (ADA) and the European Association for the Study of Diabetes (EASD) have jointly released an expert opinion-based consensus report on precision medicine [3]. The report defines precision diabetes medicine as “an approach to optimize the diagnosis, prediction, prevention, or treatment of diabetes by integrating multidimensional data, accounting for individual differences” [3], and it is characterized by six categories; precision diagnosis, precision therapeutics, precision prevention, precision treatment, precision prognosis and precision monitoring [3]. Precision medicine in diabetes utilizes the individual’s unique genetic makeup, environment or context data (that can be collected from clinical records, wearable technology, genomics and other ‘omics data) and allows one to appreciate individual characteristics, differences, circumstances and preferences [4].

For type 1 diabetes (T1D) patients, around 10% of the diabetic population, precision diagnosis integrates epidemiological data (age at diagnosis, sex, ancestry), clinical features and diagnostic test results (type of autoantibodies, genetic risk score T1D-GRS, basal and/or stimulated c-peptide measurement) to define subcategories [5]. Precision prevention for T1D considers the individual’s unique T1D risk profile (genetic susceptibility given from human leukocyte antigen *HLA* and non-*HLA* loci) to predict the individual response to the preventive agents (immune therapy or dietary intervention); however, we need to learn more about the role of environment in the onset of T1D, considering urban versus rural setting, the contribution of virus as SARS-COV2, life stressors or traumatic events, and food [6,7,8]. Precision treatment is tailored to the individual, identifying the right treatment for the right person at the right time, decreasing unwanted side effects [4]. In T1D, new long-acting insulins have improved glucose control, as well as the widespread use of continuous glucose monitoring (CGM), insulin advisors, insulin pumps and advanced hybrid closed-loop (AHCL) systems; glucose patterns derived from CGMs offer novel insights for T1D subclassification, even if we need to go beyond the glucocentric approach and also consider genetic, environmental and other individual context data.

For type 2 diabetes (T2D) subjects, clinical features, such as age at diagnosis, sex, ethnicity, BMI, HbA1c and homeostatic model assessment, can be used to subclassify clusters of individuals with insulin resistance, insulin deficiency, and lower or higher risk of complications [9]. These clinical features could predict patients who respond well to sulfonylureas, thiazolidinediones, or dipeptidyl peptidase 4 inhibitors (DPP4i); those who respond less well and those who have adverse outcomes [10,11]. There are about 12 approved classes of diabetes drugs for T2D and studies have helped to establish clinical variables that lead to individualized treatment. Instead, genetic data in T2D patients are not accurate enough to subcategorize; many genetic variants probably have a small or modest effect in the prediction of drug outcomes. The candidate gene approach has shown that few risk variants increase glycemic response to sulfonylureas or to thiazolidinediones, while others reduce the response [12,13,14,15]; genome-wide association studies (GWAS) reported variants associated with lesser or greater response to metformin [16]. Precision prevention for T2D does not consider intervening in everyone with prediabetes, because it is not cost-effective [17], but on a subset of prediabetic patients chosen on the basis of other relevant risk factors (lifestyle, socioeconomic status, family history of diabetes, ethnicity, overweight–obesity, signs of insulin resistance, genetics). A “one-size-fits-all” lifestyle intervention is not efficacious for everyone and it cannot be sustained; diet intervention and exercise programs have to be tailored, as well as risk factor exposure minimized [3].

Neonatal diabetes and maturity-onset diabetes of the young (MODY) cover 2–3% of the entire diabetic population, and precision medicine for these patients is considered as a standard of care [3]. Probabilistic algorithms or calculators that consider family history, clinical and biochemical features, have been developed to identify patients who would be candidates to be tested for monogenic diabetes by next-generation sequencing [18,19]. Precision diagnostics and treatment have an impact on the management of different forms of MODY: MODY 1 (*HNF4A*-MODY), MODY 3 (*HNF1A*-MODY) and MODY 12 (*ABCC8*-MODY) are sensitive to sulfonylureas, and precision treatment results in cessation of insulin treatment. MODY2 (*GCK*-MODY) patients do not require oral medication. Genetic diagnosis also predicts disease-related outcomes and complications; as for MODY5 (*HNF1B*-MODY) and Wolfram syndrome (*WFS1*), they should be monitored for associated disease at the kidney, liver or anything neurological, etc. Unfortunately most cases of monogenic diabetes remain misdiagnosed, mainly due to the cost of performing genetic testing [3]; other limits in implementing precision medicine in diabetes include epidemiological differences among varied populations (ethnic and racial barriers) and that some ethnic groups are underrepresented in clinical trials [20].

In conclusion, the application of precision medicine in diagnosis and in treatment of monogenic diabetes is a standard of care [3]. The same approach is nowadays not applicable to other types of diabetes: genetic and clinical data are not sufficient to predict who is more or less likely to benefit from treatment, but there is excellent potential to subcategorize the other forms of diabetes if the precision medicine approach is applied. We need to develop tools to collect and analyze patient data, and create collaborative partnerships with stakeholders (patients associations, product companies, private and public supporters of research, clinicians, educators and policy makers) that could support these projects [3]. Starting from big data, diagnostic algorithms for defining diabetes subtypes has to be developed and implemented into clinical practice, in order to offer to our patients the best therapeutic approach and follow up.
